# BOOGIE: Predicting Blood Groups from High Throughput Sequencing Data

**DOI:** 10.1371/journal.pone.0124579

**Published:** 2015-04-20

**Authors:** Manuel Giollo, Giovanni Minervini, Marta Scalzotto, Emanuela Leonardi, Carlo Ferrari, Silvio C. E. Tosatto

**Affiliations:** 1 Department of Biomedical Sciences, University of Padova, Viale G. Colombo 3, 35131 Padova, Italy; 2 Department of Information Engineering, University of Padova, Via Gradenigo 6, 35121 Padova, Italy; 3 Department of Women’s and Children’s Health, University of Padova, Padova, Italy; Seoul National University College of Medicine, KOREA, REPUBLIC OF

## Abstract

Over the last decade, we have witnessed an incredible growth in the amount of available genotype data due to high throughput sequencing (HTS) techniques. This information may be used to predict phenotypes of medical relevance, and pave the way towards personalized medicine. Blood phenotypes (e.g. ABO and Rh) are a purely genetic trait that has been extensively studied for decades, with currently over thirty known blood groups. Given the public availability of blood group data, it is of interest to predict these phenotypes from HTS data which may translate into more accurate blood typing in clinical practice. Here we propose BOOGIE, a fast predictor for the inference of blood groups from single nucleotide variant (SNV) databases. We focus on the prediction of thirty blood groups ranging from the well known ABO and Rh, to the less studied Junior or Diego. BOOGIE correctly predicted the blood group with 94% accuracy for the Personal Genome Project whole genome profiles where good quality SNV annotation was available. Additionally, our tool produces a high quality haplotype phase, which is of interest in the context of ethnicity-specific polymorphisms or traits. The versatility and simplicity of the analysis make it easily interpretable and allow easy extension of the protocol towards other phenotypes. BOOGIE can be downloaded from URL http://protein.bio.unipd.it/download/.

## Introduction

Advances in genome sequencing due to high throughput sequencing (HTS) over the last years have detected a huge amount of new Single Nucleotide Variants (SNVs) [1], producing a tremendous growth of variation databases. Finding genotype-phenotype correlations is a critical topic in personalized medicine, as personal genome sequencing is expected to become increasingly common over the next few years [2]. One of the more interesting developments in this field is the Personal Genome Project (PGP), collecting genome sequences and clinical phenotypes of participants who have signed an informed consent with the goal to make genome information freely available for research for thousands of participants [3,4]. Using this data, a number of methods have been used to predict particular phenotypes thought to be genetically determined, among which blood groups may provide a good test case.

The first discovery of blood groups dates back to the early years of the 20^th^ century, and represent an important moment for medicine. Karl Landsteiner’s ABO system was nevertheless just a first step in the characterization of blood. Forty years later the Rh was shown to play an important role, and today dozens of different blood systems have been reported [5]. As an example, the Dombrock group [6] is known to be relevant from the clinical point of view, as it can lead to severe hemolytic transfusion reactions or hemolytic disease of the newborn. Similar problems can also be caused by many other blood systems, like Lan [7], Gerebich [8] and Junior [9]. Compared to the 25 groups reported in 2000 [6], public databases currently report more than 30 blood systems [5], so it can be thought that in the close future new ones will be detected. All blood groups are determined by the presence of specific proteins on the surface of red blood cells and body fluids [10]. Their expression is fully genetically determined and cannot vary during a person’s life span. Agglutination tests have been used extensively for the identification of the ABO and Rh groups, with an error rate below 1 out of 250,000 tests [11]. On the other hand, it has been reported that non-ABO groups are among the main cause of death after blood transfusion [12], mainly due to systems like Scianna [13] where serological tests are not accurate. Sequencing techniques have been widely used as the main tool for the identification of molecular differences among systems [9,14–16]. Several opinions suggest to introduce this kind of experiment as an additional blood typing test [17,18]. Interestingly, HTS technologies have led to the widespread availability of sequencing in the medical and scientific community [19], making genetic tests more and more common for diagnostic purposes. It can be expected that genotype information will be useful for blood typing. Personal genome sequencing is expected to become increasingly used over the next few years [2], as it can predict patient response to drugs or diseases [20], and help in the diagnosis and cure. The BLOODchip system [21] is a first example of a commercial solution using genotype data to detect blood types, showing that modern sequencing techniques can be used for the identification of six different blood groups. Even though this is an appealing idea, a number of issues must still be solved, since the three billion human nucleotides are difficult to manage [22]. In addition, experimental difficulties related to sequencing and population variability [23] led to the versioning of the reference genome, and the requirement of an accurate management of SNVs linked to old genome definitions (i.e. hg18 or lesser). Last but not least, the one mutation-one phenotype paradigm used in many public databases [24,25] is clearly unable to explain many traits of clinical relevance like the ABO or Rh blood systems, where multiple co-occurring variants determine the blood group [16] [14]. The situation is finally complicated by heterozygous variants, as they require inference of the correct patient haplotype [26]. The Rh trait is just an example with good genotype knowledge and a complicated basis, since it is encoded by two different genes resulting in the two proteins RhD and RhCE [27]. The former is the determinant of the most common Rh antigen while the latter is responsible for a large part of weak inter medium Rh traits. Patients are routinely typed for D antigen and both the common terms *Rh positive* and *Rh negative* refer to the presence or absence of this antigen. The antigens C, c, E, e coded by the RhCE gene [28] are typed routinely only in patients which have developed an atypical immunological response to long-term transfusions. Furthermore, Rh is characterized by the presence of numerous hybrids resulting from genetic rearrangement of both the RhD and RhCE genes [27]. These hybrids exhibit their condition with weak serological response to the routine test. This may result in a dangerous misclassification due to both false positive and negative blood typing [29]. To complicate this scenario further, 50 other Rh system antigens are known. While these are not well studied and poorly understood, such genetic complexity well explains the importance of large human variant databases for blood groups such as BGMUT [5].

In order to improve the quality of blood typing we present BOOGIE, a method that predicts blood groups by means of a user-defined knowledge base of Boolean rules. Starting from HTS data, the tool solves the haplotype phasing problem using information stored in the user database, and uses the same data to infer the most likely blood type. BOOGIE was tested on PGP data for the prediction of 30 blood groups and their traits reported in BGMUT. This is novel, as it shows that a set of Boolean rules can predict real phenotypes, which may be complex and present a large degree of heterogeneity. Therefore, a broad range of applications can be conceived, while the increasing amount of genetic studies will significantly enhance the power of its inference engine.

## Results

BOOGIE is designed to predict phenotypes using HTS data using explicit tables that describe the correlation of SNVs with traits. These are extracted from the BGMUT database [5] which stores information about experimentally validated variants known to be relevant for determination of currently 34 different blood groups. The predictor is built to infer the closest haplotype to the known variant combinations, implicitly producing a haplotype phase. BOOGIE blood group predictions thus represent the most probable scenario of how the patient variants interact to yield a given blood group phenotype. In the following we will describe how the predictor works with the ABO group example, followed by validation on public PGP data for the ABO and Rh groups and finally presenting the distribution of predicted rarer blood groups.

### ABO case study

**Fig 1:**
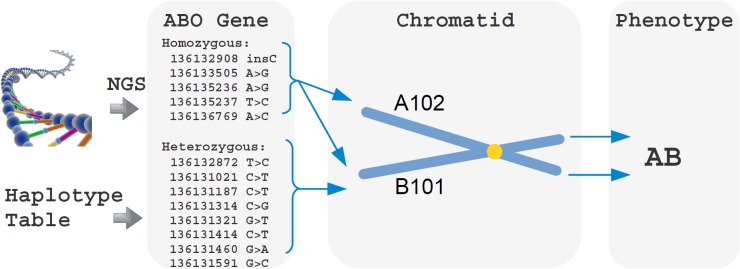
Sample ABO gene exonic mutations and phenotpe. Given the input variants, BOOGIE select all the key mutations specified in the Haplotype table for ABO group that can play a role (in chromosome 9). The tool assigns all heterozygous SNVs to the same chromatid, as this represents the most likely haplotype. As a result, the corresponding proteins will express either the A102 or B101 blood group. Genetic analysis suggests that both antigens will be present in PGP sample hu604D39, which is confirmed by a serological test. It should be noted that the SNVs reported use hg19 as reference, thus differing from the BGMUT definition of A and B blood groups. In addition, not all variants reported are necessary for the final phenotype, as some of them are common for different groups.

In order to clarify how the algorithm works, we describe the example of ABO prediction for PGP sample hu604D39 (see Fig 1), which is known to have an AB blood group. In haplotype phasing, homozygous variants are easy to manage, as they are known to appear in both chromatids. On the other hand, heterozygous ABO group SNVs can be distributed among 32 distinct allele configurations, which may produce very different traits during the final decision. Ranking the configurations by variant similarity shows that there are 3 optimal choices. All three infer a first chromatid expressing the Ax02 blood group, while the second should express either B101 or Bw19 (see Table 1). In particular, the B group allele shifts all heterozygous mutations towards a single chromatid in the best scoring configuration. This fact is well known in the literature [30] and confirms the validity of our decision strategy. Finally, the 13 SNVs in Fig 1 are fully annotated in BGMUT, yielding high confidence for our prediction. It is also interesting to note that the database contains just 99 SNVs for ABO blood group characterization. Our focus on this small set of variants seems to describe effectively even the most uncommon ABO blood groups. This is important for the efficiency as using just few decision variables rather than the full ABO sequence decreases prediction time.

**Table 1:** Sample haplotype table extracted for the ABO system.

Phenotype	Chr9:136132908	Chr9:136131650	Chr9:136131414
A101	GG	C	G
A102	GG	T	G
O02	G	C	G
B101	GG	C	A

### ABO performance

We tested BOOGIE on the PGP full genome dataset using ABO data from BGMUT. The overall accuracy is 94.2%, recognizing correctly 25/27 cases for A, 11/11 for B, 1/1 for AB and 30/32 for O group. In light of the generality of the method this is a very good result. Being able to identify the correct traits without systematic preference suggests that there is enough data in the literature to pave the way towards genetic tests for the ABO blood group. In addition, weak antigens and subgroups were identified with no effort. This is clearly an advantage compared to common serological tests and may also yield good immunological results, due to the increased ability to classify blood diversity. Regarding the amount of SNVs contained in HTS data, we observed between 4 and 18 SNVs in the genome dataset samples (see S1 Fig). Despite this large heterogeneity, BOOGIE correctly assigned heterozygous variants to the chromatid and obtained good recognition performance. It is worth to note that B typed samples typically show a higher number of exonic mutations, as also shown in past studies [30]. Interestingly, the few misclassification cases can be explained (see Table 2). In particular, profiles hu2DBF2D and hu52B7E5 were not detected as O. This is due to the ABO c.53G>T mutation, which has complete penetrance for the determination of the O group [30]. Even if this position is reported in our ABO haplotype table, our scoring system uses the same weight for all SNVs. In these three profiles, identification of the A haplotype, which was supported by 12–14 variants, was predicted to be the more likely. Of course, this could be solved from the technical point, either by adding weights to the mutations or entries to the haplotype table. Conversely, samples huFFAD87 and hu2FEC01 are quite unexpected, and can be explained by the relevance of intronic variants, and occurrence of a chromosome crossover during meiosis in the 6th intron respectively. This latter scenario is quite strange, and violates our implicit assumption of linkage disequilibrium (LD) in the coding region. It is possible that wrong variant calling or experimental errors are present, or the reported PGP participant data may simply not be correct. Three important loci for A, B or O blood group determination are in the minus strand of chromosome 9. The hg19 chromosome positions of interest are reported in the first row. The first one is a frameshift insertion, while the latter two are SNVs. With this information, we can distinguish between the B, O and two A group variants as well.

**Table 2:** Explanation for the ABO blood group mispredictions.

Profile ID	Open issue
hu2DBF2Dhu52B7E5	The highly penetrant genomic variant in ABO c.53G>T, leads to blood group O despite other variants. The other 12–14 variants of the samples suggested that A was the most likely trait. The weighting scheme or tables should be modified to deal with high impact variants.
hu2FEC01	Only three exonic ABO variants (c.220C>T, c.188 GC>AT and c.106 G>T) cannot explanation the A blood group. The group is related to (a) possible intronic variants of interest, (b) errors during the sequencing or variant calling, (c) errors during data publication on the PGP web-site.
huFFAD87	The described permutation strategy suggests that the most likely haplotype is B. A single chromosome crossover during the meiosis in intron 6 could explain the phenotype, even if this violates the expected strong linkage disequilibrium of the ABO gene. On the other hand, an incorrect report on the PGP website can be an alternative explanation.

In two cases the solution requires an improved weighting scheme, while in the latter two there is no definitive answer.

On the 23andMe dataset, BOOGIE has 91.43% accuracy. It should be noted that we removed 6 cases from the starting pool, because the experimental data had missing values for important positions. We correctly recalled 46/51 for group O, 44/44 for A, 18/21 for B and 4/5 for AB. The problem at hand is very different from the first one, due to the amount of observed SNVs (ranging from 12 to 43, as shown in S2 Fig) and data quality. In fact, it seems that a major issue for this data is identification of indel c.261delG, which is missing a in few cases, and possibly wrong in others. This would explain most of the misclassifications for this validation set. It is interesting to note that having only localized SNVs rather than complete exome data does not significantly affect performance. In fact, the ABO group is mainly determined by an accumulation of mutations related to known haplotypes. This confirms how using few localized SNVs can be effective in ABO prediction, as also assumed in previous genome-wide association studies [31]. This may have important implications for the development of commercial genetic tests for the ABO blood group, as the test of localized hot-spots can be significantly cheaper than whole exome sequencing.

### Rh performance

On the full genome data, BOOGIE has 94.2% accuracy. We recalled correctly 57/57 for Rh+ and 8/12 for Rh-. This is a good result, considering that we marked profile huC14AE1 as misclassified, where the Rh+ and Rh- scores were the same due to uncertainty in the prediction. Profile huFE71F3 and huC30901 are quite hard to explain, because they have just two SNVs in genomic coordinates 25617282 and 25634204 of chromosome 1, which seem of no impact according to the literature. A similar situation happens for profile hu025CEA.

It is interesting to note that individuals with few SNVs in the RhD gene are likely to show an Rh+ trait. In fact, almost 80% of the samples have less than five variants (see S3 Fig). Conversely, a large number of variations is related to the D-CE-D RhD hybrid group leading to a Rh- trait. CE 5-9DBT and CE 7-8-9DIVb are just two of the groups that we observed [32]. This result cannot be obtained with classical serological tests and may be relevant for highly specific blood transfusions.

On the 23andMe dataset, there are 93 Rh+ and 18 Rh- samples and our method cannot discriminate between different RhD chromatid configurations in many situations. This is mostly due to the small number of SNVs reported in 23andMe experiments (S4 Fig) which leads BOOGIE to predict Rh+ for all cases. E.g., five samples (hu3B89BD, hu8B4E43, hu25BD97, huB2C416, huF7E042) report no mutation in RhD but are marked as Rh-. On the other hand, it is well known in the literature that this trait is connected to few mutations. These 23andMe samples are clear examples where scarcity of information makes correct trait prediction based on genotype data impossible.

The coordinates reported by 23andMe experiments (c.329T>C, c.676G>C, c.712G>A, c.787G>A, c.933C>A) seem to be designed for the recognition of RhD-RhCE hybrids, but the existence of minor Rh groups (like CE 5 Va 4) makes the recognition of the correct allele configuration impossible. These positions may distinguish 32 distinct situations, while there are 67 known subgroups for Rh, leading to a non-universal assignment which cannot produce reliable results. A possible solution for this ambiguity may be to use population frequency of traits to rank tied scores. This could effectively suggest the maximum likelihood estimate in such a weakly informed context.

### Other blood groups

**Fig 2:**
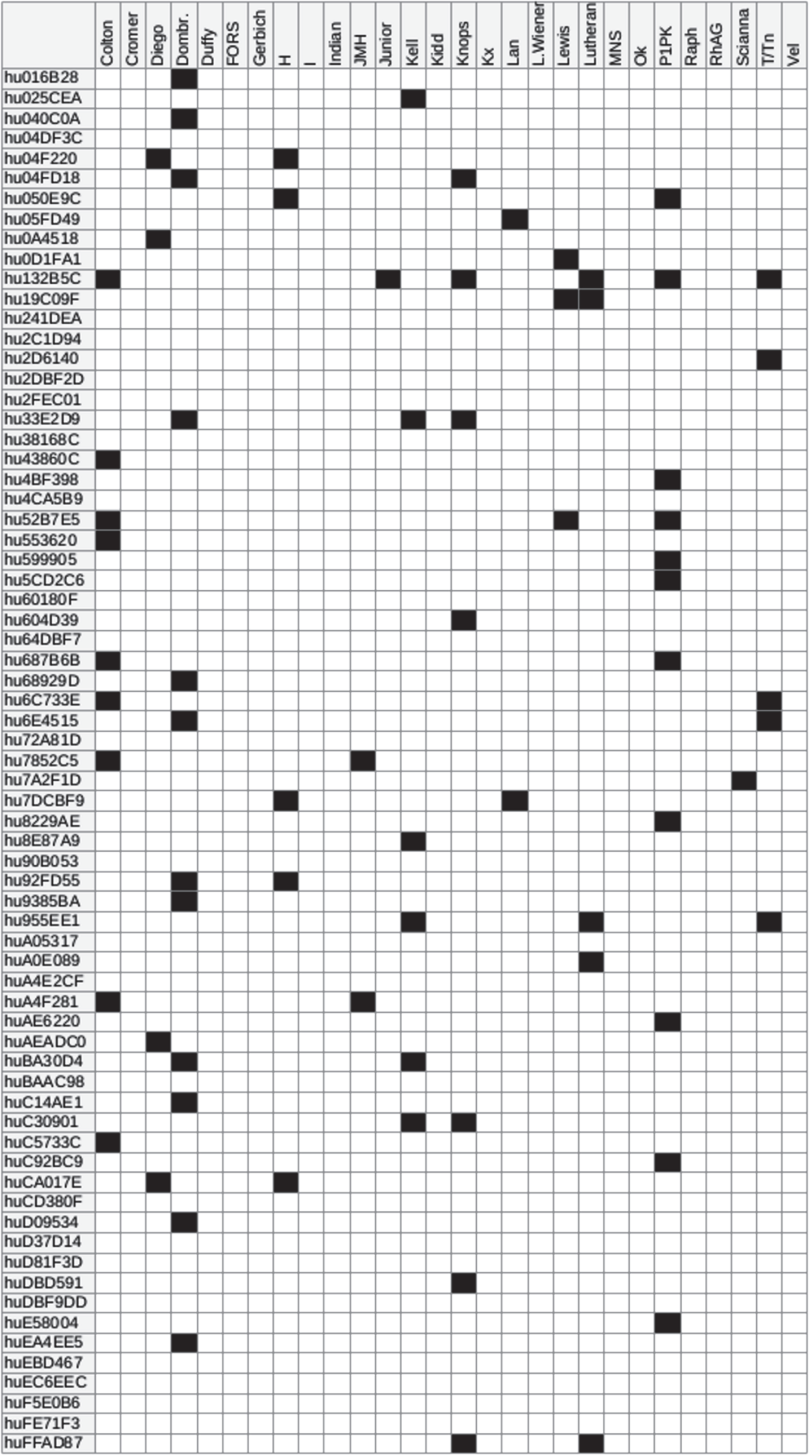
Predicted blood group distribution for the PGP full genome dataset. For each PGP sample, the possible occurrence of uncommon blood groups is highlighted. The prediction is based on the observation of coding SNVs known to be associated with uncommon blood groups. When no known variant is found, the phenotype is assumed to be the reference one. The existence of non-coding or new uncharacterized variants relevant for a blood system can influence BOOGIE, leading to some false negative predictions.

We tested the PGP genome dataset for the other 28 blood groups with BOOGIE, with striking results. As shown in Fig 2, a considerable amount of samples has uncommon traits, which may be important during blood transfusions or in related situations. For example, in the Junior system we reported the case of a weak trait. This could be relevant for hemolytic disease of the fetus and newborn, as already reported in previous studies [9]. For the Lewis and John Milton Hagen systems we observed samples with opposite traits. This is not likely to be of interest from the clinical point. Nevertheless, these groups need more study in order to completely characterize their medical relevance. It should also be noted that in the PGP web-site, no information is available for the 28 blood groups, so no validation is possible. More in general, experimental characterization of these *minor* blood-groups may currently be non trivial due to the lack of an unambiguous clinical test [18]. Routine typization is conducted with genetic analysis in patients who manifest an atypical immunological response, such as long-term transfusion patients. Methods such as BOOGIE may help to address the right strategy to adopt. Clearly, the use of highly specific annotations can be considered an additional tool to predict blood transfusion compatibility. On the other hand, it is interesting to note that HTS data can provide detailed blood group information compared to common serological tests. In fact, testing only highly specific SNVs may result in good performance, as already proven in the Rh and ABO group tests.

### Amount of non-considered mutations

**Fig 3:**
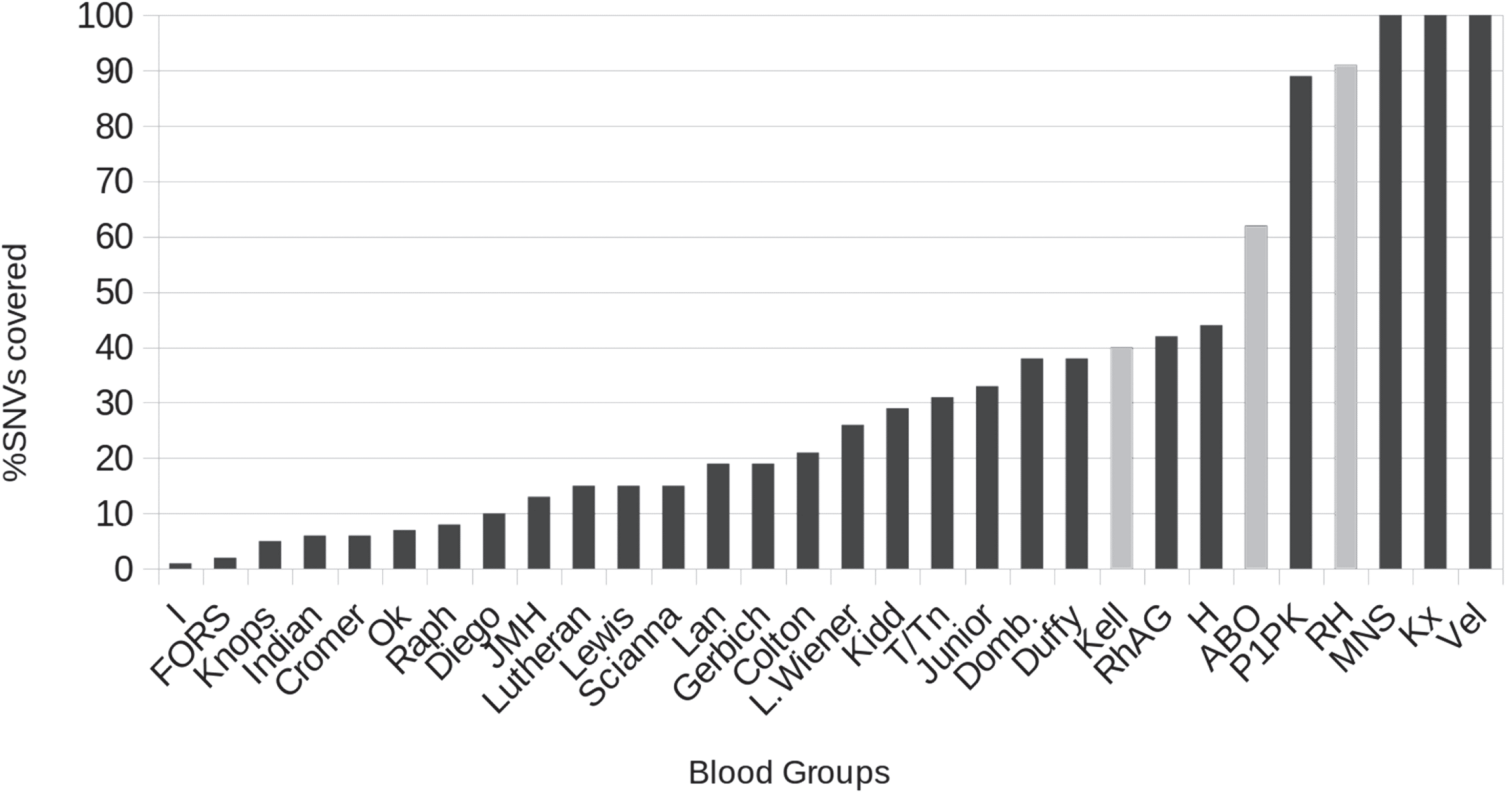
Fraction of dbSNP variants annotated for each blood group. The most important groups for the transfusion (grey bars) have good annotation in BGMUT, explaining the quality of our results. Conversely, most variants in the less studied systems still need further research for a proper automatic annotation.

Genetic variations are in many situations not relevant for the determination of a phenotype, as can be clearly seen in dbSNP. Very few mutations report a clinical relevance tag or reference to PubMed. In order to understand how much is known in the context of blood groups, we report the amount of SNVs in BGMUT and in dbSNP for each blood system, and use these two parameters to measure the completeness of our haplotype tables. Half the blood groups considered in this work seem to cover at least 20% of the SNVs in dbSNP (see Fig 3). At first glance this may seem a mixed result, but we are not interested in full coverage. We reasonably assume that (a) a small set of variants can be used as representative of a full haplotype, thanks to linkage disequilibrium and (b) a large amount of SNVs are unlikely to cause phenotypic changes. For the latter point, data from BGMUT is frequently updated, and represents the state of art for blood group characterization, so it is clear that we are using as much information as possible. As shown for the ABO and Rh systems, we argue that BOOGIE can be a valuable tool for phenotype characterization. The haplotype phase quality is also interesting and confirms that crossover recombination hot-spots are typically localized in non-coding regions [33]. This is also observed in HapMap data, where linkage disequilibrium is often observed in coding DNA. It is important in our decision as we focus only on SNVs reported in our haplotype tables, providing greater flexibility. We can either report the full gene sequence and known allele, or just describe the most relevant loci. In both situations, genes in strong linkage disequilibrium are very likely to work very well with our algorithm, as shown with ABO group tests (see S5 Fig for clarification of ABO LD). We are aware that BOOGIE will not be very effective for the less studied systems. As the amount of data available for trait prediction keeps growing (see S6 Fig), we expect our tool will become increasingly valuable over the next years. It is striking that the most of the SNVs of genes relevant for blood transfusion (RhD and ABO) are already well characterized (see Fig 3), suggesting that BOOGIE can be useful for these important blood groups.

## Discussion

In our work, we developed the tool BOOGIE for blood group prediction. The main idea is to focus on few genetic locations with annotation from the literature for a set of common phenotypes. Using this information, we resolve the haplotype phasing problem and infer the most likely trait. From the theoretical point of view, linkage disequilibrium is the key factor leading to high accuracy even in presence of a small number of observed mutations. BOOGIE was tested for human blood group prediction using PGP data and obtained very good results. Even if performance is not as good as serological tests and not yet suited for direct medical application, the few misclassification cases were easily detected. These provided interesting indications for the need of good quality SNVs in relevant spots and the importance of high penetrance mutation management, which can be easily dealt with in haplotype tables. In fact, lack of informative observations can result in the impossibility to properly classify sequencing data, as shown for the Rh group in the 23andMe dataset. Nevertheless, there is a number of advantages in our approach to be considered. The ability to directly detect ABO and Rh blood subgroups can be useful in ordinary blood transfusions. Whenever HTS data is available for some reason, it will be possible to test rare blood groups. For the 28 minor blood groups, we checked the PGP genomes for uncommon traits finding interesting results. As the experimental techniques for antigen detection are poorly sensitive in these blood systems, e.g. the Dombrock and Scianna system [13], genetic tests can be particularly useful. There is evidence of consistent discrepancies for the well-known ABO and Rh blood systems, estimated at 3.7% between real blood type and the one reported in identity documents [34]. Genetic tests could therefore represent an additional, albeit not exclusive, tool for checking compatibility during transfusions. Trait assignments in this work is based on PGP data donated by volunteers not directly connected with our laboratory. For this reason, no further experimental validation is possible for the other considered blood groups. The dataset nevertheless is clearly representative, as it contains samples of different ethnicity obtained with different experimental techniques.

BOOGIE uses a multivariate strategy based on maximum parsimony, which is particularly meaningful for proper characterization of non-trivial phenotypes, and is well explored in the context of sequence analysis. The method is very fast and produced blood group annotation for all 30 systems in a few seconds on a desktop PC. In light of the increasing amount of available HTS data, this is of great practical relevance for the quick and scalable annotation of genomes. Exponential growth of allele configurations is not a real danger, due to the limited number of heterozygous exonic SNVs typically observed in single genes. As long as the focus is on relevant hot spots, the number of permutations will be strongly reduced, leading to high computational speed. A key aspect of the system is flexibility. Simply adding more entries to the haplotype table will allow detection of new traits, while creation of new haplotype tables allows to tackle other genotype to phenotype problems. This is an important step towards the automation of trait detection in personalized medicine, in view of the constant growth of discovered phenotypes. In the context of multifactorial diseases this may be unfeasible, as we are still far from a clear description of key SNVs. Nevertheless, an increasing amount of studies may clarify these complex phenotypes and will suggest putative loci to test for mutations, as shown for cholesterol level models [35]. It should also be noted that no interpretation is possible for variants with no annotation in our knowledge base. Hence, proper reasoning will be possible only when all relevant variants are fully annotated. Dominance so far is also not considered, because it strongly depends on the context (e.g. X chromosome inactivation in women). BOOGIE computes two separate predictions, one for each chromatid. Proper interpretation of the resulting trait is left to the user and was straightforward in the ABO and Rh context. Despite these limitations, blood group traits are clearly relevant from the clinical point of view and may be effectively detected by our strategy. In addition to phenotype detection, the approach can also be a valuable tool for population studies. Some anthropological marker genes are important due to ethnicity-specific polymorphisms of certain human populations. The versatility of the tool allows us to imagine different scenarios where similar methods may be used for detection of rare diseases or in forensic medicine. In addition, BOOGIE can be downloaded (URL: http://protein.bio.unipd.it/download/) and customized for any trait prediction. Thanks to its effectiveness in HTS data interpretation, it can be of benefit for the clinical community and may help to develop of a new generation of tools for personalized medicine.

## Methods

### Data collection

In order to construct BOOGIE we had to extract as much information as possible about blood system classification from the literature. BGMUT [5] stores information about experimentally validated mutations known to be relevant for blood group determination. 34 known blood systems are described and are included in BOOGIE, with ABO and Rh of interest for method validation. The prediction strategy relies on an explicit definition of the ground truth, to be used during classification of new human samples. All loci of interest for genome classification are grouped in haplotype tables for each blood group (see S1 Table). For each known *phenotype*, the table defines all expected SNVs that should be observed for its determination. E.g. definition of the 177 known ABO blood groups uses 99 explicitly reported SNVs. Whenever BGMUT reports no data about a SNV, it is assumed to be the reference hg19 gene in our tables. Only exonic mutations were used, since they cover the largest part of the database and are more easily measurable during sequencing experiments. Obtaining the correspondence tables required meticulous manual curation, as many blood groups and traits assumed old reference genes. This is an important issue related to recent improvement of sequencing techniques, as they provided a number of different DNA sequence versions of increasing quality that cannot be easily combined. E.g. the ABO reference gene in BGMUT corresponds to the A group, while the reference gene in hg19 corresponds to the O group. This leads to a shift and re-labeling of most mutations. In many cases, like in the Indian system, SNVs simply report a wrong reference sequence, see variations in [36]. In addition, HTS data after variant calling uses genomic coordinates rather than gene-based coordinates. For this reason we used the BiomaRt [37] R package for quick coordinate translation, and manually fixed all mismatches in case of discrepancies with hg19. All conflicting cases were solved using the public databases dbSNP [24], UCSC reference genes [38], Phencode [39] and the original publications from PubMed. We decided to drop traits and mutations when the manual conversion failed. After this process, we obtained annotation for more than 800 different traits based on almost 1,000 unique coding variants for 30 blood groups (see S1 Table for more details).

### Phenotype prediction

In HTS experiments, data obtained after variant calling provides an invaluable source of information for phenotype prediction. On the other hand, heterozygous mutations by themselves do not explicitly specify the genomic sample haplotype. This is known as the haplotype phasing problem in the literature. It can be solved effectively using information from HapMap [40] and expectation maximization approaches with Hidden Markov Models [41]. These methods are computationally expensive and based on low resolution data, which cannot distinguish rare haplotypes or uncommon SNVs [40]. Due to these limitations, we developed a new fast tool that could deal with (a) haplotype phasing and (b) phenotype decisions for the resulting predicted chromatid. For a particular patient gene, we assumed without loss of generality that there are N homozygous and M heterozygous mutations. With no prior assumption, this would lead to 2^M-1^ possible allele configurations *C* in the two human chromatids. As a first step, we enumerated all configurations, where exactly one of them represents reality. In the second step, we solved jointly the haplotype phasing and phenotype decision problem using our haplotype tables *H*. Given a particular configuration *c*, we determined its phenotype by means of a K-nearest neighbour strategy using data in the haplotype table *H*. In other words, we measured the sequence distance of *c* to those in H, and transferred by similarity the phenotype of the closest. Similarity is measured as Hamming distance, i.e. number of substitutions and insertions between two sequences, which is also important for determination of the most likely haplotype phase. The higher the value, the lesser the chance to observe *c* in nature. This is clearly linked to the maximum parsimony principle, which is well established in phylogenetic reconstruction [42]. We ranked all allele configurations by Hamming distance and selected the best as real allele configuration. The overall idea is that we are trying to find an example in our haplotype table which has the same mutations, so we can be reasonably confident that phenotype and haplotype will be the same. Conversely, a wrong allele configuration will look very different from the previously published ones and is not likely to exist at all.

### BOOGIE system

**Fig 4:**
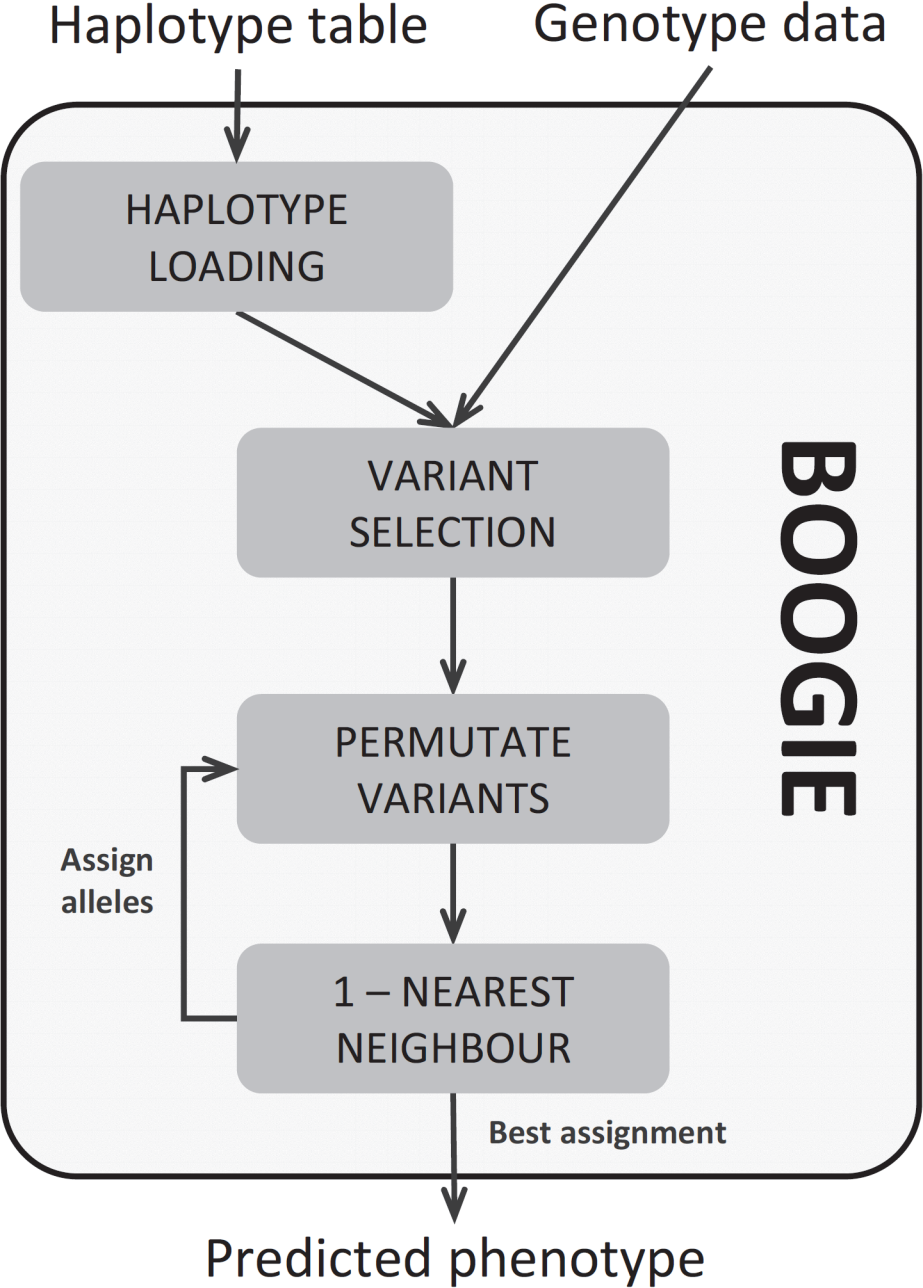
Schematic BOOGIE overview. The tool requires just genotype data and an haplotype table for its execution. Genotype must be specified in a tabular file similar to VCF format, where chromosome, genomic position, nucleotides ad zygosity are specified. Haplotypes are defined in a tabular file, and each row specify the expected SNVs of a target phenotype. See README file of the application for format details. BOOGIE search for key variants in the input genotype, and optimize their assignment to a haplotypes with known phenotype according to the 1-nearest neighbour algorithm. The SNV permutation with best score is the one with highest phenotype likelihood.

BOOGIE was written with Java JDK 1.7, making it portable to all major operating systems. As shown in Fig 4, BOOGIE requires two files for its execution. The haplotype table for the phenotype of interest and the target genotype file. Format details are provided in a README file. Variants contained in the genotype file will be considered if and only if they are part of the haplotype table, i.e., they are useful for phenotype prediction. Once the variants have been selected, all the possible assignment to the two human chromatids are enumerated. The phenotype of each permutation is predicted by means of the 1-nearest neighbour algorithm, and the corresponding score of the most similar haplotype stored. The two assignments with overall maximal score become the predicted phenotypes. Note that dominance is not taken into account, so it is up to the user to determine the final traits. E.g., if the two alleles A and 0 are predicted for the ABO blood group, the expert user should infer that the A group is dominant.

### PGP dataset

The first goal for testing our method was the collection of samples with phenotype annotation and genetic data. We chose to work with public data from the PGP [3,4], mainly due to the richness of the clinical profiles. From the 2,651 profiles accessed on 13 May 2013, entries having ABO and Rh annotation with sequencing data were extracted. We obtained two datasets with 69 samples with full genome sequences from Complete Genomics and 111 with 23andMe SNP data. The nature of the 23andMe data set is very heterogeneous in array size (ranging from 570k to 1000k SNVs) and chip type (customized Illumina Hap550+ or HumanOmniExpress BeadChip Kit). The reference genome used was either hg18 or hg19. For a detailed description of the data, please refer to the PGP website (URL: http://personalgenomes.org/). The full genome dataset was used for benchmarking the accuracy of the tool for ABO and Rh when full data is available. The 23andMe dataset was used as a further set to evaluate our prediction strategy when only partial information is available.

## Supporting Information

S1 Fig Frequency of samples having between 4 and 18 SNVs in the ABO gene the for the PGP full genome set. (DOC)

S2 Fig Frequency of samples in the 23andMe dataset for the ABO gene. (DOC)

S3 Fig Sample frequency based on RHD gene SNVs for the PGP full genome dataset. (DOC)

S4 Fig Sample frequency in the 23andMe dataset for the RHD gene. (DOC)

S5 Fig HapMap reports that the ABO gene is in strong linkage disequilibrium (LD) (DOC)

S6 Fig The number of blood traits reported in the literature is growing linearly. (DOC)

S1 Table Summary of BGMUT data. (DOC)

## References

[1] Altshuler DM, Gibbs RA, Peltonen L, Dermitzakis E, Schaffner SF, Yu F, et al Integrating common and rare genetic variation in diverse human populations. Nature. 2010;467: 52–58. 10.1038/nature09298 20811451 PMC3173859

[2] Bubnoff A. Next-Generation Sequencing: The Race Is On. Cell. 2008;132: 721–723. 10.1016/j.cell.2008.02.028 18329356

[3] Ball MP, Thakuria JV, Zaranek AW, Clegg T, Rosenbaum AM, Wu X, et al A public resource facilitating clinical use of genomes. Proc Natl Acad Sci USA. 2012;109: 11920–11927. 10.1073/pnas.1201904109 22797899 PMC3409785

[4] Church GM. The Personal Genome Project. Mol Syst Biol. 2005;1 10.1038/msb4100040 PMC1681452 16729065

[5] Patnaik SK, Helmberg W, Blumenfeld OO. BGMUT: NCBI dbRBC database of allelic variations of genes encoding antigens of blood group systems. Nucleic Acids Res. 2012;40: D1023–1029. 10.1093/nar/gkr958 22084196 PMC3245102

[6] Gubin AN, Njoroge JM, Wojda U, Pack SD, Rios M, Reid ME, et al Identification of the Dombrock blood group glycoprotein as a polymorphic member of the ADP-ribosyltransferase gene family. Blood. 2000;96: 2621–2627. 11001920

[7] Helias V, Saison C, Ballif BA, Peyrard T, Takahashi J, Takahashi H, et al The human porphyrin transporter ABCB6 is dispensable for erythropoiesis but responsible for the new blood group system Langereis. Nat Genet. 2012;44: 170–173. 10.1038/ng.1069 22246506 PMC3664204

[8] Colin Y, Rahuel C, London J, Roméo PH, d’ Auriol L, Galibert F, et al Isolation of cDNA clones and complete amino acid sequence of human erythrocyte glycophorin C. J Biol Chem. 1986;261: 229–233. 2416746

[9] Saison C, Helias V, Ballif BA, Peyrard T, Puy H, Miyazaki T, et al Null alleles of ABCG2 encoding the breast cancer resistance protein define the new blood group system Junior. Nat Genet. 2012;44: 174–177. 10.1038/ng.1070 22246505 PMC3653631

[10] Denomme GA. Molecular basis of blood group expression. Transfusion and Apheresis Science. 2011;44: 53–63. 10.1016/j.transci.2010.12.010 21277830

[11] Goodnough LT, Brecher ME, Kanter MH, AuBuchon JP. Transfusion Medicine—Blood Transfusion. New England Journal of Medicine. 1999;340: 438–447. 10.1056/NEJM199902113400606 9971869

[12] Goodnough LT, Levy JH, Murphy MF. Concepts of blood transfusion in adults. Lancet. 2013;381: 1845–1854. 10.1016/S0140-6736(13)60650-9 23706801

[13] Van der Schoot CE, Veldhuisen B, de Haas M. Will Genotyping Replace Serology in Future Routine Blood Grouping?—Opinion 5. Transfus Med Hemother. 2009;36: 234–235. 10.1159/000214840 21113269 PMC2980536

[14] Döscher A, Vogt C, Bittner R, Gerdes I, Petershofen EK, Wagner FF. RHCE alleles detected after weak and/or discrepant results in automated Rh blood grouping of blood donors in Northern Germany. Transfusion. 2009;49: 1803–1811. 10.1111/j.1537-2995.2009.02221.x 19453979

[15] Lublin DM, Kompelli S, Storry Jr, Reid ME. Molecular basis of Cromer blood group antigens. Transfusion. 2000;40: 208–213. 10.1046/j.1537-2995.2000.40020208.x 10686005

[16] Yip SP. Sequence variation at the human ABO locus. Annals of Human Genetics. 2002;66: 1–27. 10.1017/S0003480001008995 12014997

[17] Anstee DJ. Red cell genotyping and the future of pretransfusion testing. Blood. 2009;114: 248–256. 10.1182/blood-2008-11-146860 19411635

[18] Storry JR, Olsson ML. Will Genotyping Replace Serology in Future Routine Blood Grouping?—Opinion 4. Transfus Med Hemother. 2009;36: 232–233. 10.1159/000214939 21113268 PMC2980535

[19] Mardis ER. The impact of next-generation sequencing technology on genetics. Trends in Genetics. 2008;24: 133–141. 10.1016/j.tig.2007.12.007 18262675 PMC2680276

[20] Burke W, Psaty BM. PErsonalized medicine in the era of genomics. JAMA. 2007;298: 1682–1684. 10.1001/jama.298.14.1682 17925520

[21] Avent ND, Martinez A, Flegel WA, Olsson ML, Scott ML, Nogués N, et al The Bloodgen Project of the European Union, 2003–2009. Transfus Med Hemother. 2009;36: 162–167. 10.1159/000218192 21113258 PMC2980524

[22] Cooper GM, Shendure J. Needles in stacks of needles: finding disease-causal variants in a wealth of genomic data. Nat Rev Genet. 2011;12: 628–640. 10.1038/nrg3046 21850043

[23] Iafrate AJ, Feuk L, Rivera MN, Listewnik ML, Donahoe PK, Qi Y, et al Detection of large-scale variation in the human genome. Nat Genet. 2004;36: 949–951. 10.1038/ng1416 15286789

[24] Sherry ST, Ward M-H, Kholodov M, Baker J, Phan L, Smigielski EM, et al dbSNP: the NCBI database of genetic variation. Nucleic Acids Res. 2001;29: 308–311. 11125122 10.1093/nar/29.1.308 PMC29783

[25] Horaitis O, Talbot CC, Phommarinh M, Phillips KM, Cotton RGH. A database of locus-specific databases. Nat Genet. 2007;39: 425–425. 10.1038/ng0407-425 17392794

[26] Frazer KA, Ballinger DG, Cox DR, Hinds DA, Stuve LL, Gibbs RA, et al A second generation human haplotype map of over 3.1 million SNPs. Nature. 2007;449: 851–861. 10.1038/nature06258 17943122 PMC2689609

[27] Flegel WA. Molecular genetics and clinical applications for RH. Transfusion and Apheresis Science. 2011;44: 81–91. 10.1016/j.transci.2010.12.013 21277262 PMC3042511

[28] Bugert P, Scharberg EA, Geisen C, von Zabern I, Flegel WA. RhCE protein variants in Southwestern Germany detected by serologic routine testing. Transfusion. 2009;49: 1793–1802. 10.1111/j.1537-2995.2009.02220.x 19453980 PMC5314459

[29] Legler TJ, Eber SW, Lakomek M, Lynen R, Maas JH, Pekrun A, et al Application of RHD and RHCE genotyping for correct blood group determination in chronically transfused patients. Transfusion. 1999;39: 852–855. 10504121 10.1046/j.1537-2995.1999.39080852.x

[30] Amado M, Bennett EP, Carneiro F, Clausen H. Characterization of the Histo-Blood Group O2 Gene and Its Protein Product. Vox Sanguinis. 2000;79: 219–226. 10.1046/j.1423-0410.2000.7940219.x 11155073

[31] Burton PR, Clayton DG, Cardon LR, Craddock N, Deloukas P, Duncanson A, et al Genome-wide association study of 14,000 cases of seven common diseases and 3,000 shared controls. Nature. 2007;447: 661–678. 10.1038/nature05911 17554300 PMC2719288

[32] Huang CH, Chen Y, Reid ME, Okubo Y. Evidence for a separate genetic origin of the partial D phenotype DBT in a Japanese family. Transfusion. 1999;39: 1259–1265. 10.1046/j.1537-2995.1999.39111259.x 10604255

[33] Kauppi L, Jeffreys AJ, Keeney S. Where the crossovers are: recombination distributions in mammals. Nat Rev Genet. 2004;5: 413–424. 10.1038/nrg1346 15153994

[34] Rentas FJ, Clark PA. Blood type discrepancies on military identification cards and tags: a readiness concern in the U.S. Army. Mil Med. 1999;164: 785–787. 10578589

[35] Pas NCA van de, Woutersen RA, Ommen B van, Rietjens IMCM, Graaf AA de. A physiologically based in silico kinetic model predicting plasma cholesterol concentrations in humans. J Lipid Res. 2012;53: 2734–2746. 10.1194/jlr.M031930 23024287 PMC3494263

[36] Telen MJ, Udani M, Washington MK, Levesque MC, Lloyd E, Rao N. A Blood Group-related Polymorphism of CD44 Abolishes a Hyaluronan-binding Consensus Sequence without Preventing Hyaluronan Binding. J Biol Chem. 1996;271: 7147–7153. 10.1074/jbc.271.12.7147 8636151

[37] Kasprzyk A. BioMart: driving a paradigm change in biological data management. Database. 2011;2011: bar049–bar049. 10.1093/database/bar049 22083790 PMC3215098

[38] Meyer LR, Zweig AS, Hinrichs AS, Karolchik D, Kuhn RM, Wong M, et al The UCSC Genome Browser database: extensions and updates 2013. Nucleic Acids Research. 2012;41: D64–D69. 10.1093/nar/gks1048 23155063 PMC3531082

[39] Giardine B, Riemer C, Hefferon T, Thomas D, Hsu F, Zielenski J, et al PhenCode: connecting ENCODE data with mutations and phenotype. Human Mutation. 2007;28: 554–562. 10.1002/humu.20484 17326095

[40] Thorisson GA, Smith AV, Krishnan L, Stein LD. The International HapMap Project Web site. Genome Res. 2005;15: 1592–1593. 10.1101/gr.4413105 16251469 PMC1310647

[41] Browning SR, Browning BL. Rapid and Accurate Haplotype Phasing and Missing-Data Inference for Whole-Genome Association Studies By Use of Localized Haplotype Clustering. The American Journal of Human Genetics. 2007;81: 1084–1097. 10.1086/521987 17924348 PMC2265661

[42] Tamura K, Peterson D, Peterson N, Stecher G, Nei M, Kumar S. MEGA5: Molecular Evolutionary Genetics Analysis Using Maximum Likelihood, Evolutionary Distance, and Maximum Parsimony Methods. Molecular Biology and Evolution. 2011;28: 2731–2739. 10.1093/molbev/msr121 21546353 PMC3203626

