# Supplementary material for: BOOGIE: Predicting Blood Groups from High Throughput Sequencing Data
Source: PLoS One. 2015 Apr 20;10(4):e0124579. doi: 10.1371/journal.pone.0124579 (PMC4404330; doi:10.1371/journal.pone.0124579)
Supplement: S1 Fig — (DOC) [file pone.0124579.s001.doc]

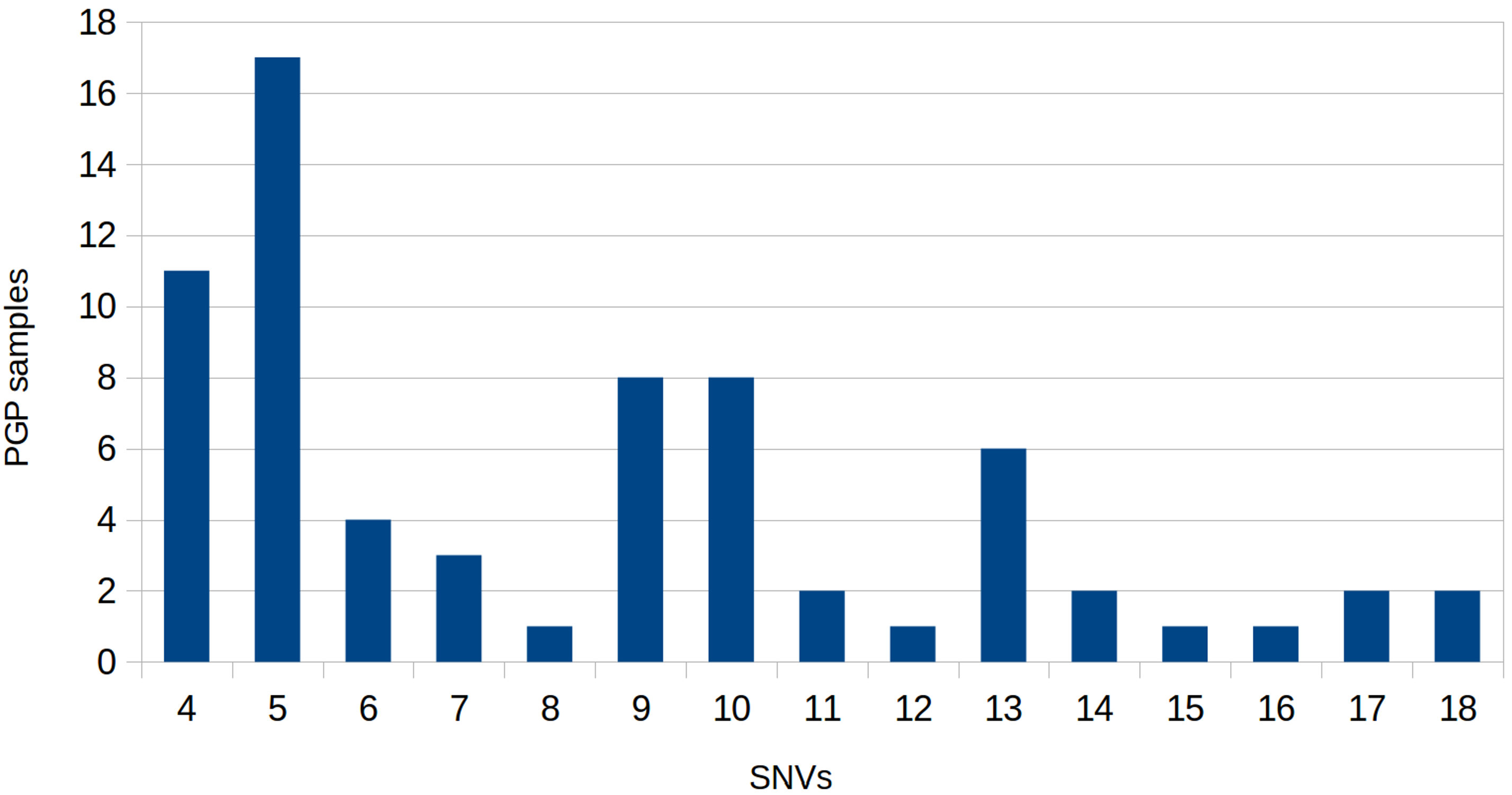


**S1 Figure.** **Frequency of samples having between 4 and 18 SNVs in the ABO gene the for the PGP full genome set.** Almost half of the individuals have between 4 and 5 mutations. This is clearly connected to the O blood group. Conversely, the amount of people with more than 10 variants is smaller and related to the B blood group. Regardless of the amount of variants, the permutation-matching strategy is able to recover the correct phenotype.
