# Supplementary material for: BOOGIE: Predicting Blood Groups from High Throughput Sequencing Data
Source: PLoS One. 2015 Apr 20;10(4):e0124579. doi: 10.1371/journal.pone.0124579 (PMC4404330; doi:10.1371/journal.pone.0124579)
Supplement: S2 Fig — (DOC) [file pone.0124579.s002.doc]

**
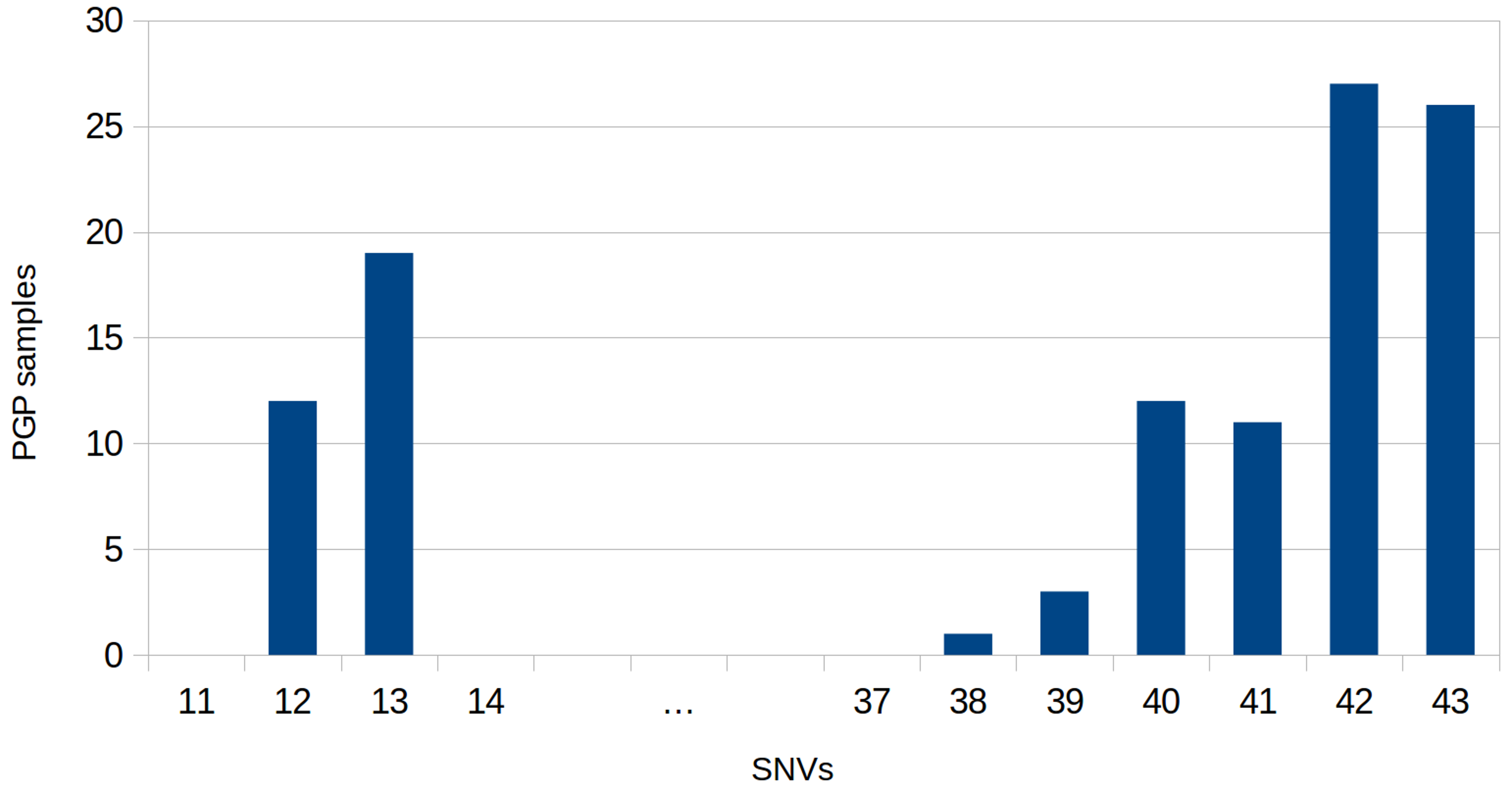
**

**S2 Figure. Frequency of samples in the 23andMe dataset for the ABO gene.** There is a heterogeneous amount of patient variants. In fact, one third of the samples report 12-13 mutations, while the rest contain more than 38 SNVs.
