# Supplementary material for: BOOGIE: Predicting Blood Groups from High Throughput Sequencing Data
Source: PLoS One. 2015 Apr 20;10(4):e0124579. doi: 10.1371/journal.pone.0124579 (PMC4404330; doi:10.1371/journal.pone.0124579)
Supplement: S3 Fig — (DOC) [file pone.0124579.s003.doc]

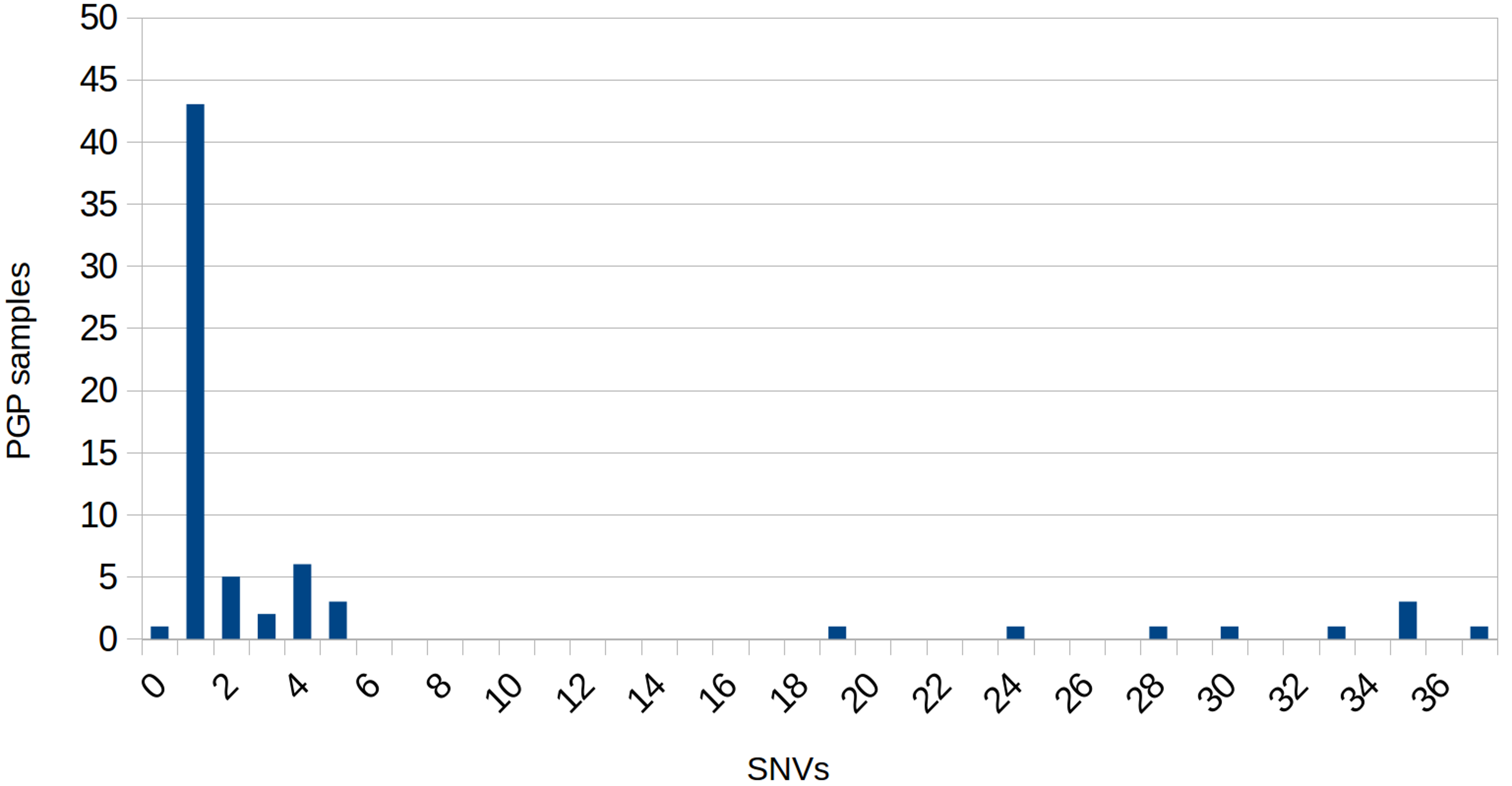


**S3 Figure. Sample frequency based on RHD gene SNVs for the PGP full genome dataset.** Almost 90% of individuals (the ones with Rh+) have less than 6 mutations. The rest of the participants are good candidates for Rh-.
