# Supplementary material for: BOOGIE: Predicting Blood Groups from High Throughput Sequencing Data
Source: PLoS One. 2015 Apr 20;10(4):e0124579. doi: 10.1371/journal.pone.0124579 (PMC4404330; doi:10.1371/journal.pone.0124579)
Supplement: S4 Fig — (DOC) [file pone.0124579.s004.doc]

**
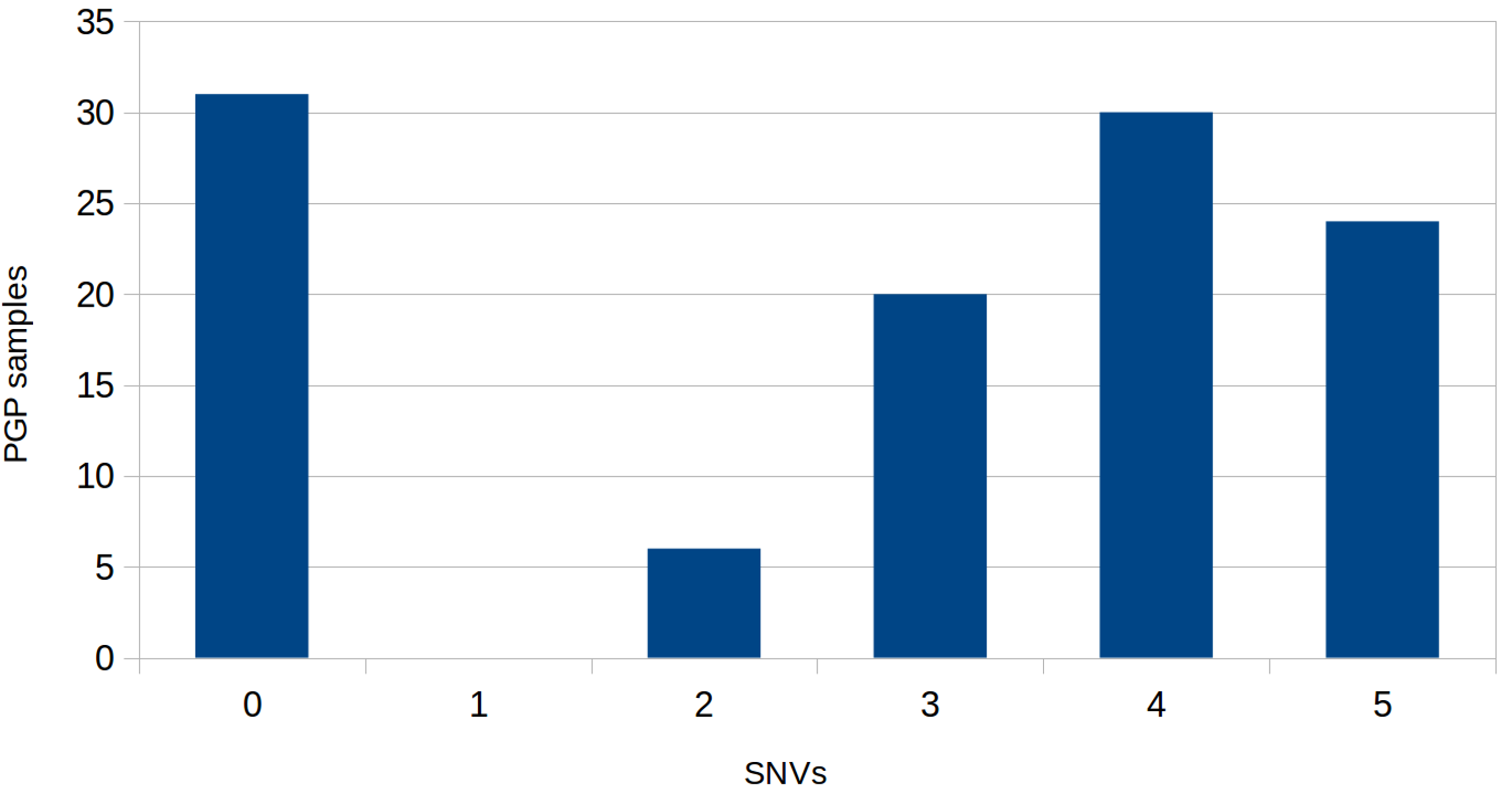
**

**S4 Figure. Sample frequency in the 23andMe dataset for the RHD gene.** A small number of variants is observed. One third of the participants reports no mutations at all, while the rest of the samples have at most 5 mutations.
