# Supplementary material for: BOOGIE: Predicting Blood Groups from High Throughput Sequencing Data
Source: PLoS One. 2015 Apr 20;10(4):e0124579. doi: 10.1371/journal.pone.0124579 (PMC4404330; doi:10.1371/journal.pone.0124579)
Supplement: S5 Fig — (DOC) [file pone.0124579.s005.doc]

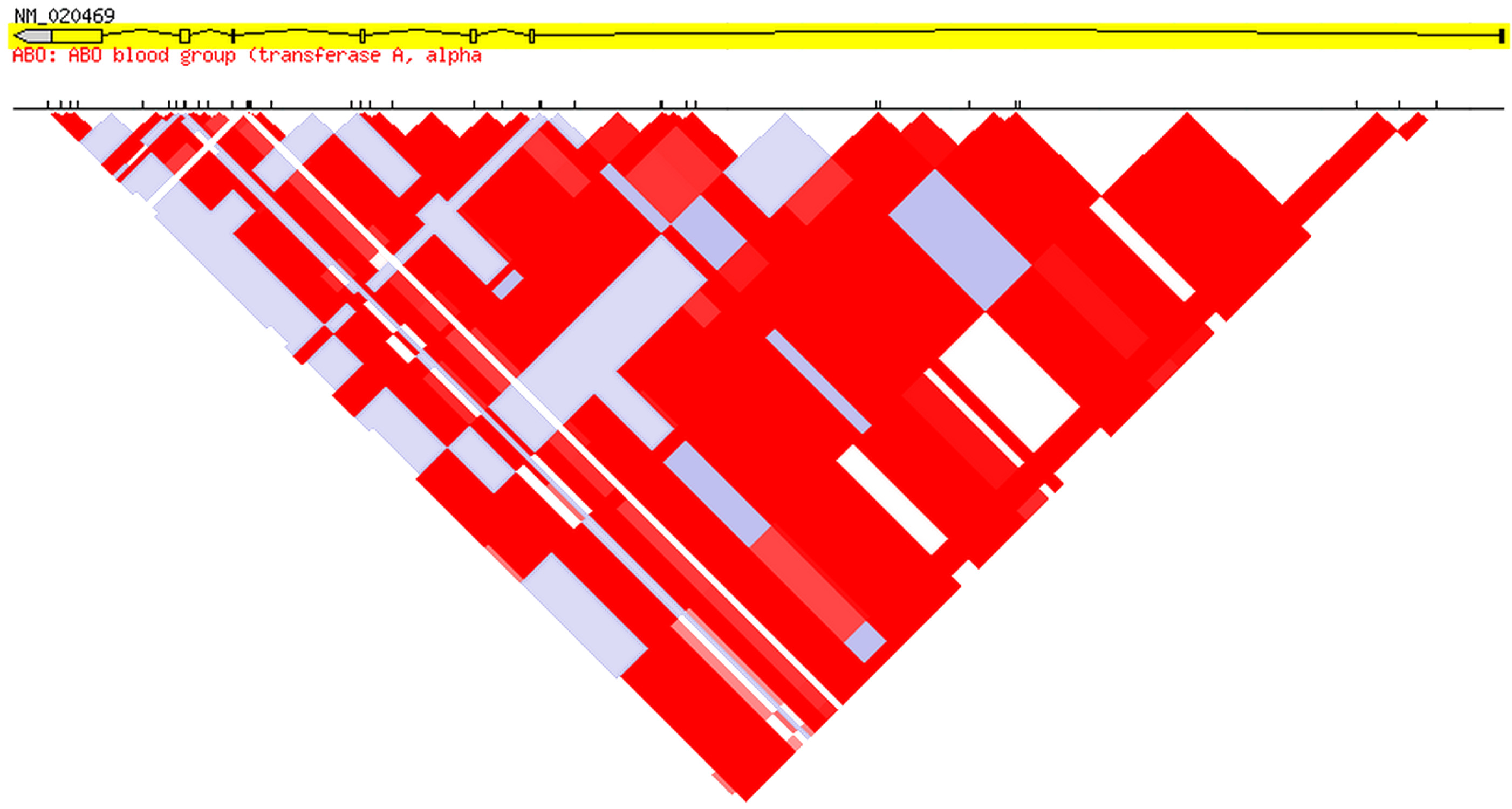


**S5 Figure. HapMap reports that the ABO gene is in strong linkage disequilibrium (LD).**  In fact, the tagged SNVs across the ABO sequence, show high LD (displayed in red) for the entire gene. Conversely, intergenetic regions have much lower LD (in white). Therefore, using even few mutations, it is possible to reconstruct the entire gene structure. This confirms that the haplotype phasing strategy used in BOOGIE can effectively determine a correct phase.
