# Supplementary material for: BOOGIE: Predicting Blood Groups from High Throughput Sequencing Data
Source: PLoS One. 2015 Apr 20;10(4):e0124579. doi: 10.1371/journal.pone.0124579 (PMC4404330; doi:10.1371/journal.pone.0124579)
Supplement: S6 Fig — (DOC) [file pone.0124579.s006.doc]

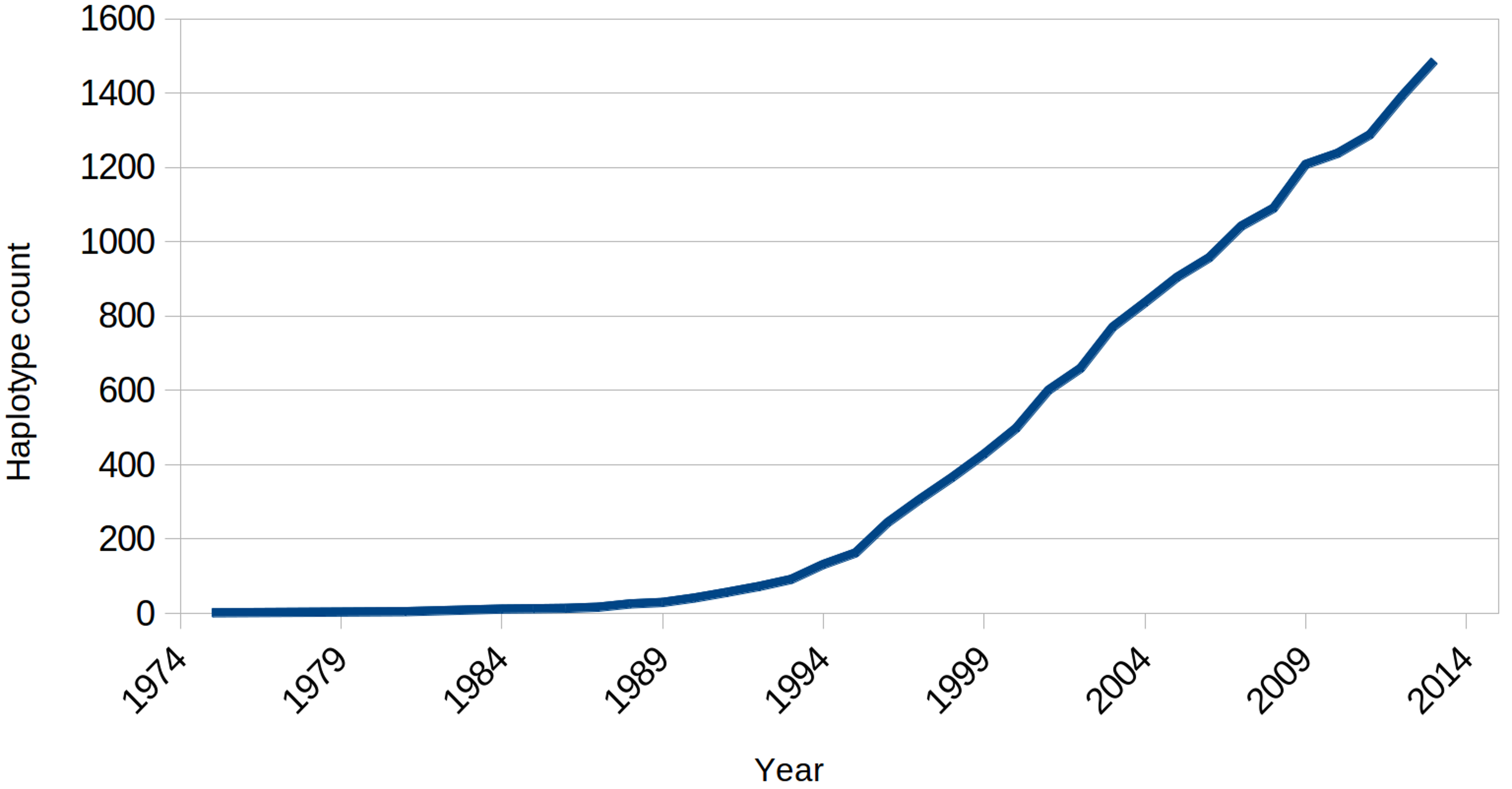


**S6 Figure. The number of blood traits reported in the literature is growing linearly.** The development of sequencing techniques in the nineties enables the detection of new blood phenotypes. This quick growth is important for tools like BOOGIE, as new data can improve prediction performance and allows the study of additional phenotypes.
