# Supplementary material for: BOOGIE: Predicting Blood Groups from High Throughput Sequencing Data
Source: PLoS One. 2015 Apr 20;10(4):e0124579. doi: 10.1371/journal.pone.0124579 (PMC4404330; doi:10.1371/journal.pone.0124579)
Supplement: S1 Table — (DOC) [file pone.0124579.s007.doc]

| **Blood System** | **Reference** | **Traits** | **SNVs** | **dbSNP** | **unused SNVs** |
| --- | --- | --- | --- | --- | --- |
| ABO | (Yip 2000) | 177 | 100 | 112 | 4 |
| Colton | (Preston et al. 1994) | 10 | 9 | 43 | 4 |
| Cromer | (Lublin et al. 2000) | 15 | 13 | 241 | 2 |
| Diego | (Jarolim et al. 1998) | 26 | 23 | 231 | 9 |
| Dombrock | (Gubin et al. 2000) | 16 | 18 | 48 | 1 |
| Duffy | (Rios et al. 2000) | 10 | 21 | 56 | 3 |
| FORS | (Svensson et al. 2013) | 2 | 1 | 70 | 2 |
| Gerbich | (Colin et al. 1986) | 6 | 6 | 34 | 3 |
| H | (Larsen et al. 1990) | 97 | 98 | 160 | 1 |
| I | (Yeh et al. 1999) | 9 | 7 | 783 | 3 |
| Indian | (Harn et al. 1991) | 3 | 6 | 113 | 10 |
| JMH | (Yamada et al. 1999) | 11 | 11 | 86 | 5 |
| Junior | (Saison et al. 2012) | 19 | 31 | 96 | 2 |
| Kell | (Lee et al. 1997) | 56 | 56 | 136 | 3 |
| Kidd | (Inoue et al. 2004) | 17 | 16 | 56 | 3 |
| Knops | (Moulds et al. 2001) | 7 | 6 | 185 | 3 |
| Kx | (Danek et al. 2001) | 18 | 34 | 19 | 0 |
| Lan | (Helias et al. 2012) | 14 | 21 | 123 | 0 |
| L-Wiener | (Hermand et al. 1995) | 3 | 11 | 44 | 1 |
| Lewis | (Soejima et al. 2009) | 47 | 34 | 234 | 2 |
| Lutheran | (Crew et al. 2003) | 19 | 25 | 175 | 11 |
| MNS | (Huang and Blumenfeld 1991) | 28 | 108 | 72 | 4 |
| Ok | (Spring et al. 1997) | 5 | 6 | 97 | 3 |
| P1PK | (Hellberg et al. 2003) | 43 | 69 | 77 | 3 |
| Raph | (Crew et al. 2004) | 5 | 3 | 40 | 2 |
| RhAG | (Cherif-Zahar et al. 1996) | 17 | 20 | 46 | 4 |
| RH | (Döscher et al. 2009) | 113 | 166 | 166 | 8 |
| Scianna | (Wagner et al. 2003) | 8 | 11 | 74 | 3 |
| T/Tn | (Ju et al. 2002) | 13 | 13 | 43 | 0 |
| Vel | (Cvejic et al. 2013) | 2 | 17 | 5 | 0 |

**S1 Table. Summary of BGMUT data.** The Traits column corresponds to the number of phenotypes stored in our haplotype tables, while SNVs specifies the number of decisional variables accounted for the blood group. dbSNP reports the number of exonic mutations known for the genes involved in the blood system. Finally, unused SNVs shows how many exon mutations cannot be interpreted in the PGP full genome dataset due to missing annotation in public databases.

**References**

Cherif-Zahar,B. *et al.* (1996) Candidate gene acting as a suppressor of the RH locus in most cases of Rh-deficiency. *Nat. Genet.*, **12**, 168–173.

Colin,Y. *et al.* (1986) Isolation of cDNA clones and complete amino acid sequence of human erythrocyte glycophorin C. *J. Biol. Chem.*, **261**, 229–233.

Crew,V.K. *et al.* (2004) CD151, the first member of the tetraspanin (TM4) superfamily detected on erythrocytes, is essential for the correct assembly of human basement membranes in kidney and skin. *Blood*, **104**, 2217–2223.

Crew,V.K. *et al.* (2003) Molecular bases of the antigens of the Lutheran blood group system. *Transfusion (Paris)*, **43**, 1729–1737.

Cvejic,A. *et al.* (2013) SMIM1 underlies the Vel blood group and influences red blood cell traits. *Nat. Genet.*, **45**, 542–545.

Danek,A. *et al.* (2001) McLeod neuroacanthocytosis: genotype and phenotype. *Ann. Neurol.*, **50**, 755–764.

Döscher,A. *et al.* (2009) RHCE alleles detected after weak and/or discrepant results in automated Rh blood grouping of blood donors in Northern Germany. *Transfusion (Paris)*, **49**, 1803–1811.

Gubin,A.N. *et al.* (2000) Identification of the Dombrock blood group glycoprotein as a polymorphic member of the ADP-ribosyltransferase gene family. *Blood*, **96**, 2621–2627.

Harn,H.-J. *et al.* (1991) The multispecific cell adhesion molecule CD44 is represented in reticulocyte cDNA. *Biochem. Biophys. Res. Commun.*, **178**, 1127–1134.

Helias,V. *et al.* (2012) The human porphyrin transporter ABCB6 is dispensable for erythropoiesis but responsible for the new blood group system Langereis. *Nat. Genet.*, **44**, 170–173.

Hellberg,Å. *et al.* (2003) Additional molecular bases of the clinically important p blood group phenotype. *Transfusion (Paris)*, **43**, 899–907.

Hermand,P. *et al.* (1995) Molecular basis and expression of the LWa/LWb blood group polymorphism. *Blood*, **86**, 1590–1594.

Huang,C.H. and Blumenfeld,O.O. (1991) Identification of recombination events resulting in three hybrid genes encoding human MiV, MiV(J.L.), and Sta glycophorins. *Blood*, **77**, 1813–1820.

Inoue,H. *et al.* (2004) Identification and characterization of a Kidd antigen/UT-B urea transporter expressed in human colon. *Am. J. Physiol. - Cell Physiol.*, **287**, C30–C35.

Jarolim,P. *et al.* (1998) Characterization of Seven Low Incidence Blood Group Antigens Carried by Erythrocyte Band 3 Protein Presented in part at the 49th Annual Meeting of the American Association of Blood Banks, Orlando, FL, October 6-10, 1996. *Blood*, **92**, 4836–4843.

Ju,T. *et al.* (2002) Cloning and Expression of Human Core 1 β1,3-Galactosyltransferase. *J. Biol. Chem.*, **277**, 178–186.

Larsen,R.D. *et al.* (1990) Molecular cloning, sequence, and expression of a human GDP-L-fucose:beta-D-galactoside 2-alpha-L-fucosyltransferase cDNA that can form the H blood group antigen. *Proc. Natl. Acad. Sci.*, **87**, 6674–6678.

Lee,S. *et al.* (1997) Molecular basis for the high-incidence antigens of the Kell blood group system. *Transfusion (Paris)*, **37**, 1117–1122.

Lublin,D. m. *et al.* (2000) Molecular basis of Cromer blood group antigens. *Transfusion (Paris)*, **40**, 208–213.

Moulds,J.M. *et al.* (2001) Molecular identification of Knops blood group polymorphisms found in long homologous region D of complement receptor 1. *Blood*, **97**, 2879–2885.

Preston,G.M. *et al.* (1994) Mutations in aquaporin-1 in phenotypically normal humans without functional CHIP water channels. *Science*, **265**, 1585–1587.

Rios *et al.* (2000) New genotypes in Fy(a− b−) individuals: nonsense mutations (Trp to stop) in the coding sequence of either FY A or FY B. *Br. J. Haematol.*, **108**, 448–454.

Saison,C. *et al.* (2012) Null alleles of ABCG2 encoding the breast cancer resistance protein define the new blood group system Junior. *Nat. Genet.*, **44**, 174–177.

Soejima,M. *et al.* (2009) Genetic variation of FUT3 in Ghanaians, Caucasians, and Mongolians. *Transfusion (Paris)*, **49**, 959–966.

Spring,F.A. *et al.* (1997) The Oka blood group antigen is a marker for the M6 leukocyte activation antigen, the human homolog of OX-47 antigen, basigin and neurothelin, an immunoglobulin superfamily molecule that is widely expressed in human cells and tissues. *Eur. J. Immunol.*, **27**, 891–897.

Svensson,L. *et al.* (2013) Forssman expression on human erythrocytes: biochemical and genetic evidence of a new histo-blood group system. *Blood*, **121**, 1459–1468.

Wagner,F.F. *et al.* (2003) Scianna antigens including Rd are expressed by ERMAP. *Blood*, **101**, 752–757.

Yamada,A. *et al.* (1999) Molecular Cloning of a Glycosylphosphatidylinositol-Anchored Molecule CDw108. *J. Immunol.*, **162**, 4094–4100.

Yeh,J.-C. *et al.* (1999) Molecular Cloning and Expression of a Novel β-1,6-N-Acetylglucosaminyltransferase That Forms Core 2, Core 4, and I Branches. *J. Biol. Chem.*, **274**, 3215–3221.

Yip,S.P. (2000) Single-tube multiplex PCR-SSCP analysis distinguishes 7 common ABO alleles and readily identifies new alleles. *Blood*, **95**, 1487–1492.
